# Lymphoblastoid Cell Lines as a Tool to Study Inter-Individual Differences in the Response to Glucose

**DOI:** 10.1371/journal.pone.0160504

**Published:** 2016-08-10

**Authors:** Michael A. Grassi, Vidhya R. Rao, Siquan Chen, Dingcai Cao, Xiaoyu Gao, Patricia A. Cleary, R. Stephanie Huang, Andrew D. Paterson, Rama Natarajan, Jalees Rehman, Timothy S. Kern, DCCT/EDIC Research Group

**Affiliations:** 1 Department of Ophthalmology & Visual Sciences, University of Illinois at Chicago, Chicago, Illinois, United States of America; 2 Cellular Screening Center, Institute for Genomics and Systems Biology, University of Chicago, Chicago, Illinois, United States of America; 3 The Biostatistics Center, George Washington University, Rockville, Maryland, United States of America; 4 Department of Medicine, Section of Hematology/Oncology, University of Chicago, Chicago, Illinois, United States of America; 5 Genetics and Genome Biology Research Institute, Sickkids, Dalla Lana School of Public Health, University of Toronto, Toronto, Canada; 6 Department of Diabetes Complications and Metabolism, Beckman Research Institute of the City of Hope, Duarte, California, United States of America; 7 Department of Pharmacology, University of Illinois at Chicago, Chicago, Illinois, United States of America; 8 Departments of Medicine and Pharmacology Case Western Reserve University, Cleveland, Ohio, United States of America, and the Veterans Administration Medical Center Research Service 151, Cleveland, Ohio, United States of America; Wayne State University, UNITED STATES

## Abstract

**Background:**

White blood cells have been shown in animal studies to play a central role in the pathogenesis of diabetic retinopathy. Lymphoblastoid cells are immortalized EBV-transformed primary B-cell leukocytes that have been extensively used as a model for conditions in which white blood cells play a primary role. The purpose of this study was to investigate whether lymphoblastoid cell lines, by retaining many of the key features of primary leukocytes, can be induced with glucose to demonstrate relevant biological responses to those found in diabetic retinopathy.

**Methods:**

Lymphoblastoid cell lines were obtained from twenty-three human subjects. Differences between high and standard glucose conditions were assessed for expression, endothelial adhesion, and reactive oxygen species.

**Results:**

Collectively, stimulation of the lymphoblastoid cell lines with high glucose demonstrated corresponding changes on molecular, cellular and functional levels. Lymphoblastoid cell lines up-regulated expression of a panel of genes associated with the leukocyte-mediated inflammation found in diabetic retinopathy that include: a cytokine (*IL-1B* fold change = 2.11, p-value = 0.02), an enzyme (*PKCB* fold change = 2.30, p-value = 0.01), transcription factors *(NFKB-p50* fold change = 2.05, p-value = 0.01), *(NFKB-p65* fold change = 2.82, p-value = 0.003), and an adhesion molecule (*CD18* fold change = 2.59, 0.02). Protein expression of CD18 was also increased (p-value = 2.14x10^-5^). The lymphoblastoid cell lines demonstrated increased adhesiveness to endothelial cells (p = 1.28x10^-5^). Reactive oxygen species were increased (p = 2.56x10^-6^). Significant inter-individual variation among the lymphoblastoid cell lines in these responses was evident (F = 18.70, p < 0.0001).

**Conclusions:**

Exposure of lymphoblastoid cell lines derived from different human subjects to high glucose demonstrated differential and heterogeneous gene expression, adhesion, and cellular effects that recapitulated features found in the diabetic state. Lymphoblastoid cells may represent a useful tool to guide an individualized understanding of the development and potential treatment of diabetic complications like retinopathy.

## Introduction

A significant barrier to progress in the treatment of diabetic retinopathy is that it is a complex, multifactorial condition caused by the interactions of multiple genetic and environmental components. This has resulted in only marginal progress by our group and others in defining its key underlying molecular elements [1–6]. For instance, targeting the angiogenic factor, VEGF, has enjoyed considerable success in treating manifestations of diabetic retinopathy in some but not all patients suggesting heterogeneous underlying etiologies [7]. Novel approaches that facilitate an individualized understanding of mechanisms and possible therapeutic strategies for this condition are urgently needed. A potential way to advance care for diabetic complications like retinopathy is to molecularly characterize disease-relevant tissue from large numbers of diabetic human subjects who have been longitudinally followed for decades.

Pre-existing lymphoblastoid cell lines are available for thousands of subjects from several landmark clinical studies of diabetes whose depth, scope and duration may never be repeated. Lymphoblastoid cell lines are immortalized EBV-transformed primary B-cell leukocytes. Lymphoblastoid cells maintain primary leukocyte features for many inflammatory and genetic conditions [8]. For instance, we have previously shown that lymphoblastoid cells preserve their inter-individual variation in adhesion to endothelial cells, an important leukocyte property in retinopathy [9]. Lymphoblastoid cell lines have been shown to be relevant not only to white blood cells but also to a diverse array of different tissues [8, 10–12]. Recent findings of the GTEx study confirm the substantial overlap in the genetic architecture for gene expression between lymphoblastoid cells and other tissues [13, 14].

Accordingly, we hypothesized that the individual molecular response to glucose should be maintained in lymphoblastoid cells. In this proof of principle study, we specifically tested whether distinct lymphoblastoid cell lines could be stimulated with chronic high glucose exposure to demonstrate heterogeneous and differential expression, adhesion, and cellular effects.

## Methods

### Subject Safety and Confidentiality Issues

All subject cell lines were de-identified prior to their arrival at the University of Illinois at Chicago; therefore, this proposal qualified as nonhuman subjects research according to the guidelines set forth by the Institutional Review Board at the University of Illinois at Chicago. As the data were analyzed anonymously, no subject consent was required. The analyses performed at George Washington University did not involve protected health information as the phenotypic data was de-identified. The George Washington University institutional review board has approved all analyses of EDIC data of this nature. Specific approval for this study was obtained from the EDIC Research Review Committee. All protocols used for this portion of the study are in accordance with federal regulations and the principles expressed in the Declaration of Helsinki.

### Cell Lines

Twenty-three lymphoblastoid cell lines were used in the study.

Sixteen of the lymphoblastoid cell lines were generated from individuals with type 1 diabetes from the DCCT/EDIC cohort (labeled with the de-identified subject numbers of 1–16 in data tables). The Diabetes Control and Complications Trial (DCCT) was a multi-center randomized clinical trial that demonstrated the benefit of intensive glycemic management in reducing the risk of development and progression of diabetic retinal and other microvascular complications in patients with type 1 diabetes [15]. Follow-up of DCCT cohort was continued in the Epidemiology of Diabetes Interventions and Complications (EDIC) observational study [16]. Whole blood samples were ascertained from the study subjects between 1991 and 1993. White blood cells from study subjects were extracted, processed and frozen at the University of Minnesota in the Central Biochemistry Laboratory, where they were transformed into lymphoblastoid cell lines in the early 2000s. The sixteen diabetic lymphoblastoid cell lines used in this study were obtained from the EDIC repository at the Central Biochemistry Laboratory.

The sixteen lymphoblastoid cell lines from diabetic subjects consisted of eight pairs of matched cases and controls. Cases were defined by the development of proliferative diabetic retinopathy by EDIC Year 10, whereas controls had no retinopathy through EDIC Year 10. Most subjects were matched by age, gender, treatment group (intensive vs. conventional), cohort (primary vs. secondary), and diabetes duration (S1 Table)[17, 18], but it was not possible to match all eight pairs in this fashion; therefore, 1 pair was matched by age, gender and treatment group only. Diabetes duration was defined as the number of months since the onset of diabetes at DCCT baseline which was the time at subject enrollment (1983–1989). Experiments were conducted in a masked fashion in order to reduce any bias prior to the analysis.

The remaining seven lymphoblastoid cell lines were purchased from the Coriell Institute for Medical Research NIGMS Human Genetic Cell Repository (http://ccr.coriell.org/) (GM14581, GM14569, GM14381, GM07012, GM14520, GM11985, and GM07344). All of these subjects were included in one of our prior published studies [9]. None of these subjects had a history diabetes. Both male and female subjects were included. All of these control subjects were unrelated and of Caucasian ethnicity (S2 Table).

Lymphoblastoid cell lines at each site were established using standard Epstein-Barr virus (EBV) transformation protocols.

### Culture Conditions

All lymphoblastoid cells were maintained in conventional lymphocyte cell culture conditions of RPMI 1640 with 10% FBS in a 25-cm^2^cell culture flask. The cells were incubated at 37^0^ C in 5% CO_2_ and the media was changed twice each week. Prior to the experiments (below), lymphoblastoid cells following serum starvation were passaged for a minimum of one week in either standard RPMI 1640 (11 mM glucose) or high glucose RPMI media (30mM glucose) [19].

### mRNA Expression

Gene expression was assessed for a panel of genes that have previously been implicated in the pathogenesis of diabetic retinopathy through leukocyte-mediated mechanisms of inflammation. The panel included: *TNF alpha [20–23], IL-1 beta [24–27], NFKB-p50 [28, 29], NFKB-p65 [30], CD18 [31, 32], PKCB [33–35]*, and *GAPDH*. Pre-validated Prime Time qPCR primers (IDT Coralville, Iowa, USA) (S3 Table) for each gene were used. *GAPDH* was used as internal control for all cell lines [36]. Total RNA was isolated from lymphoblastoid cell lines using TRIzol reagent (Invitrogen, Carlsbad, CA). cDNA was synthesized using the High-Capacity cDNA Reverse Transcription Kit (Life Technologies, Grand Island, NY, USA). qRTPCR was performed by ABI Prism 7900 (Applied Biosystems, Foster City, CA, USA) using power SYBR Green PCR master mix (Life Technologies, Grand Island, NY, USA). qPCR amplifications were performed for 40 cycles of denaturation at 95°C for 10 seconds, annealing/extension 60°C for 30 seconds. The melting curves were generated to detect the melting temperatures of the products following the PCR run. The relative mRNA levels were determined by the comparative CT method [37] with the fold change calculated using the equation 2^-Δ(Δct)^.

### Protein expression of CD18

Lymphoblastoid cells were collected in 15 mL tubes and counted using Countess (Life Technologies). 200,000 cells from each sample were transferred into U bottom 96 well plates. The cells were pelleted at 300g at 4°C (Eppendorf Bench top with 96 well plate adaptor). The pelleted cells were washed with 0.2 mL of ice cold PBS (1X, Gibco, Life Technologies, Grand Island, NY) three times. The cells were then suspended in 5% heat inactivated serum for 30 minutes. Cells were washed with ice cold PBS the cells and then incubated with FITC conjugated Anti-Human CD18 (BD Biosciences, San Jose, CA) for 1 hour in 1% BSA containing the antibody titer as indicated by the manufacturer. FITC conjugated IgG alone served as negative control. The CD18 labeled cells were washed with ice cold PBS and suspended in ice cold PBS. The CD18 expression was measured by flow cytometry using the BD LSRFortessa™ cell analyzer and the data were analyzed at the UIC flow cytometry core facility, Chicago, IL.

### Leukocyte endothelial adhesion

Lymphoblastoid cell lines were plated on a monolayer of human retinal microvascular endothelial cells (HRMEC) (ACBRI 181, Cell Systems, Kirkland, WA) to test their adhesion as we have described previously [9]. HRMECs were maintained in MCDB 131 medium (5.5 mM glucose) containing 10% fetal calf serum (FCS, Invitrogen, Life Technologies, Carlsbad, CA), 100 IU/ ml penicillin, 100 μg/ml streptomycin, and 0.25 μg/ml amphotericin B supplemented with 1 μg/ml epidermal growth factor and 10 μg/ml hydrocortisone (complete medium) as we have done previously [36]. All experiments using HRMECs were performed between passages 5 and 8. HRMECs were counted using the Beckman coulter counter, plated at a density of 30,000 cells/well in flat clear bottom black 96-well plates (Corning, Acton, MA), and cultured to confluency.

On the day of the assay, lymphoblastoid cells at a concentration of 1×10^6^ cells/mL were incubated with 2 μM Calcein AM (BD Biosciences, Bedford, MA) for 30 minutes at 37°C. The cells were collected and subjected to three washes with PBS to remove the free Calcein AM. Calcein AM labeled cells were added at a density of 50,000 cells/well on top of confluent monolayer cultures of HRMEC in 96-well plates and incubated for 30 minutes at 37°C. Non-adherent cells were removed by an optimized automated wash protocol adapted using the EP3 liquid handling system. Calcein AM fluorescence in the labeled cells was assessed by the high content Acumen imager at an excitation and emission wavelength of 485/535 nm before and after each wash.

Leukocyte endothelial adhesion was assessed for each lymphoblastoid cell line 4x (1 column of four wells) by 1) measuring raw fluorescence for both the pre-wash and post-wash 2) converting the relative fluorescence units (RFUs) to cell number using a standard curve 3) calculating lymphoblastoid cell line % remaining post-wash and 4) normalizing cell % based on plate controls to account for interplate variability. A mean, standard deviation, and coefficient of variation (CV) for each lymphoblastoid cell line were calculated. A z-score and CV for each plate were determined. Differences in leukocyte endothelial adhesion between lymphoblastoid cell lines and the control lines were determined in order to calculate the fold change as a metric of comparison for each lymphoblastoid cell line.

### Reactive Oxygen Species (ROS)

1 million cells/mL (5mL) were added to T-25 flasks and grown in standard and high glucose conditions. Following treatment, cellular hydroxyl radical (-OH) was detected using the oxidation-sensitive fluorescent dye, CMH2DCFDA (488 nm excitation/535 nm emission) (Life Technologies, Grand Island, NY)). Cells treated with 25 μM H_2_O_2_ served as the positive control for ROS measurement. (1μM) pre-dissolved CMH2DCFDA in PBS was then added to the cells and incubated at 37°C for 30 minutes. After incubation, the cells were washed with PBS and suspended in PBS. The fluroescence was measured by flow cytometry using the BD LSRFortessa™ cell analyzer and the data were analyzed at the UIC flow cytometry core facility, Chicago, IL. The gated geometric mean value (r) was used for comparing ROS expression in samples.

### Statistical Analyses

Data for gene expression are presented as mean with 95% confidence intervals. Statistical differences between groups were analyzed with a standard two-tailed paired Student *t* test using Microsoft Excel software. A p-value of less than 0.05 was considered statistically significant.

Inter-individual differences among the lymphoblastoid cell lines were assessed using a one-way ANOVA, with the differences in gene expression mediated by high glucose as the outcome. Internal consistency among gene expressions was quantified as the Cronbach alpha.

Gene expression and subject covariates were used to determine the principal components. Variance components were calculated using a mixed model. The covariates of age, gender, treatment group, cohort, diabetes duration, and case/control status were included in the model as random effects. High glucose exposure was treated as a fixed effect and a repeated measure. The covariance given by the restricted maximum likelihood (REML) is used in the calculation for the proportion of treatment effect [38]. Age was defined as a binary variable from < = 30 years (N = 7) and >30 (N = 9) at DCCT baseline. Duration is defined as a binary variable from < = 36 months (N = 8) and >36 (N = 8) at DCCT baseline.

## Results

### High glucose induces up-regulation of gene expression in lymphoblastoid cells

**Fig 1:**
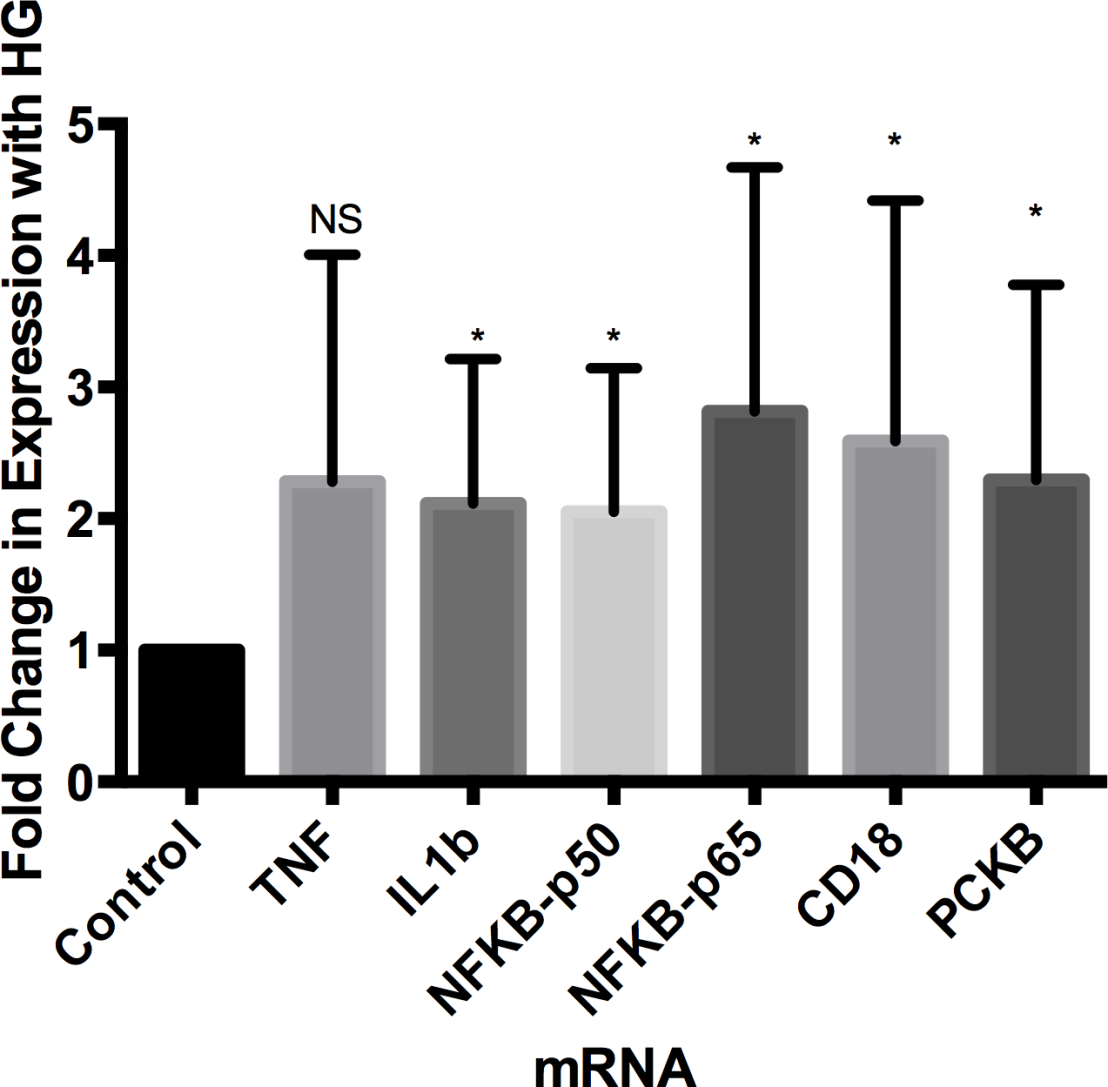
High glucose induces gene expression up-regulation in lymphoblastoid cell lines. Diabetes associated genes are increased in lymphoblastoid cell lines (n = 23) exposed to high glucose (HG) (30 mM). Figure is a bar graph of the fold change in gene expression in response to high glucose. Expression was normalized to *GAPDH*. Change in gene expression is based on the difference in expression of each gene under high and standard glucose conditions (11 mM). Error bars represent 95% confidence intervals of fold change. * p-value < 0.05. NS Not significant p > 0.05.

Differential fold change in mRNA expression of a panel of six genes previously implicated in the leukocyte associated inflammation of diabetic retinopathy was determined in twenty-three lymphoblastoid cell lines. Fold change was calculated based on a comparison of expression for that cell line under high (30 mM) and standard (11 mM) glucose conditions. Specifically, for each cell line gene expression was compared under high (HG Δct) and standard glucose (SG Δct) conditions to determine the differential glucose mediated expression for that cell line (Δ(Δct)) (S4 Table). All six genes demonstrated a > 2 mean fold change induction in high glucose (range 2.05–2.82) (Fig 1). Significant results were identified that included: *IL-1B* (fold change = 2.11, p-value = 0.02), *NFKB-p50* (fold change = 2.05, p-value = 0.01), *NFKB-p65* (fold change = 2.82, p-value = 0.003), *CD18* (fold change = 2.59, 0.02), and *PKCB* (fold change = 2.30, p-value = 0.01). The only exception was *TNF-alpha*, which at a p-value of 0.06, showed a trend towards significance but did not meet the threshold. The control *GAPDH* gene did not show any change with high glucose (p-value = 0.35). Among the cell lines, induction of gene expression in the six assessed genes in response to high glucose was significantly correlated (r = 0.40–0.94, Cronbach alpha = 0.95).

### Dynamic stimulation of lymphoblastoid cell lines with glucose reveals significant inter-individual differences

Differences in gene expression under high glucose conditions were compared among the twenty-three cell lines (S4 Table). We were interested in whether each line generated a unique response to the fixed stimulus of 30mM glucose. We hypothesized that induction of the lines with a uniform stimulus would generate a differential response due to the underlying genomic differences among cell lines as each line is generated from a different individual. In order to minimize confounding factors, we confirmed that no difference in viability was evident for the cell lines at the study conditions of 11 mM and 30 mM glucose. Analysis of variance of the differences in gene expression mediated by high glucose among the cell lines was conducted. Significant inter-individual differences were found among the lymphoblastoid cell lines (F = 18.70, p < 0.0001), with 78% of overall variance in glucose-mediated gene expression change accounted for by the use of different lymphoblastoid cell lines. Hence, transformation and multiple freeze/thaw passages do not appear to homogenize the individual response in gene expression to high glucose in lymphoblastoid cell lines. Rather, the major factor that determines differences in gene expression to high glucose stimulation is individual dependent. In order to identify the covariate that plays the primary role in determining differences among subjects in their response to glucose, variance component analysis was conducted which similarly identified the individual response as the key factor (S1 Fig). It is unlikely that the greater number of female subjects affects the study findings as gender was not found to be a significant confounding covariate either in the variance analysis or in the principal component analysis. This is consistent with prior epidemiologic studies that have failed to find an association between gender and the development or progression of diabetic retinopathy. Thus, it appears that with provocation through high glucose exposure, lymphoblastoid cell lines lose their homogenous, uniform behavior [39], and reveal underlying unique responses that may be characteristic of the individual from which they were generated.

Principal component analysis of the twenty-three samples based on gene expression did not reveal clustering of any of the three groups of subjects (proliferative diabetic retinopathy (PDR), diabetes without retinopathy (No PDR), and no diabetes (No DM)) (S2 Fig). As a quality measure this was reassuring as it suggests that there was not a major difference between the groups in any potentially confounding factors like culture conditions, the transformation process, or demographic features. Similar to the ANOVA, the principal component analysis suggested that most of the variance (73.9%) was explained by individual subject differential response to high glucose.

### High glucose increases the protein expression of CD18

**Fig 2:**
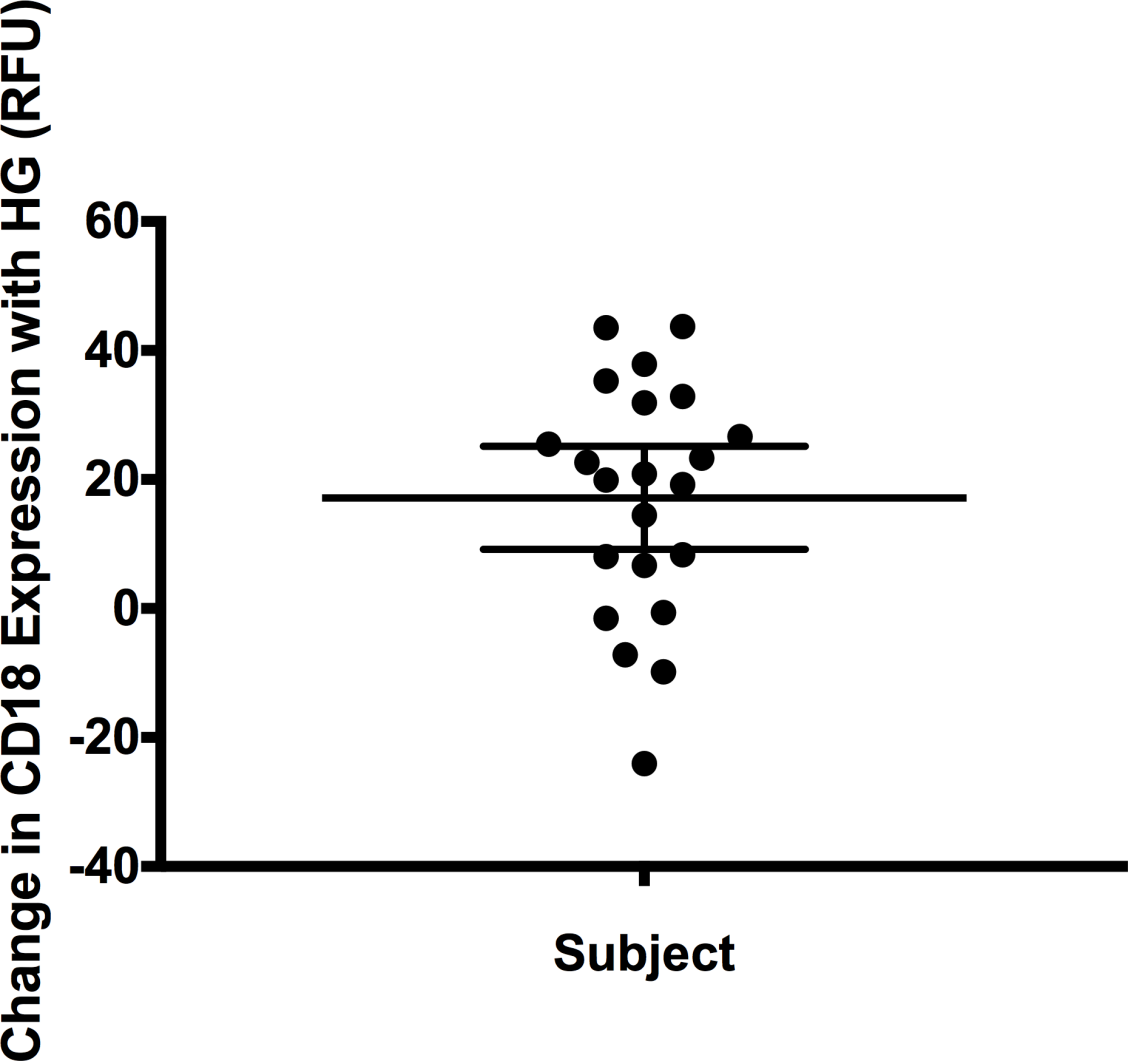
Expression of CD18 by flow cytometry is increased in lymphoblastoid cell lines exposed to high glucose. CD18 expression was compared between standard glucose (11 mM) and high glucose (HG) (30 mM) conditions. CD18 is increased in high glucose (p = 2.14x10^-5^) consistent with the mRNA expression findings. Figure is a univariate scatter plot showing the distribution of change in relative fluorescence units (RFU) for CD18 expression in high glucose compared to standard glucose conditions in twenty-two lymphoblastoid cell lines. Most but not all cell lines demonstrate an increase in CD18 under high glucose conditions. Mean and 95% confidence intervals shown.

CD18, a leukocyte cell surface adhesion molecule that binds with ICAM-1 implicated in the pathogenesis of diabetic retinopathy [32], was assessed by flow cytometry using an antibody-based fluorescence assay. Expression was determined for twenty-three lymphoblastoid cell lines under standard and high glucose conditions (S5 Table). One of the cell lines had to be removed from the analysis (total n = 22), as the flow cytometer did not calculate a value for it under standard glucose conditions. Consistent with the gene expression findings for *CD18*, the flow cytometry revealed an increase in the expression of CD18 with high glucose (p-value = 2.14x10^-5^) (Fig 2). Within a cell line the values of the mRNA and protein expression for CD18 were not significantly correlated, perhaps due to the small sample size and inherent experimental noise in these assays.

### High glucose increases leukocyte endothelial adhesion

**Fig 3:**
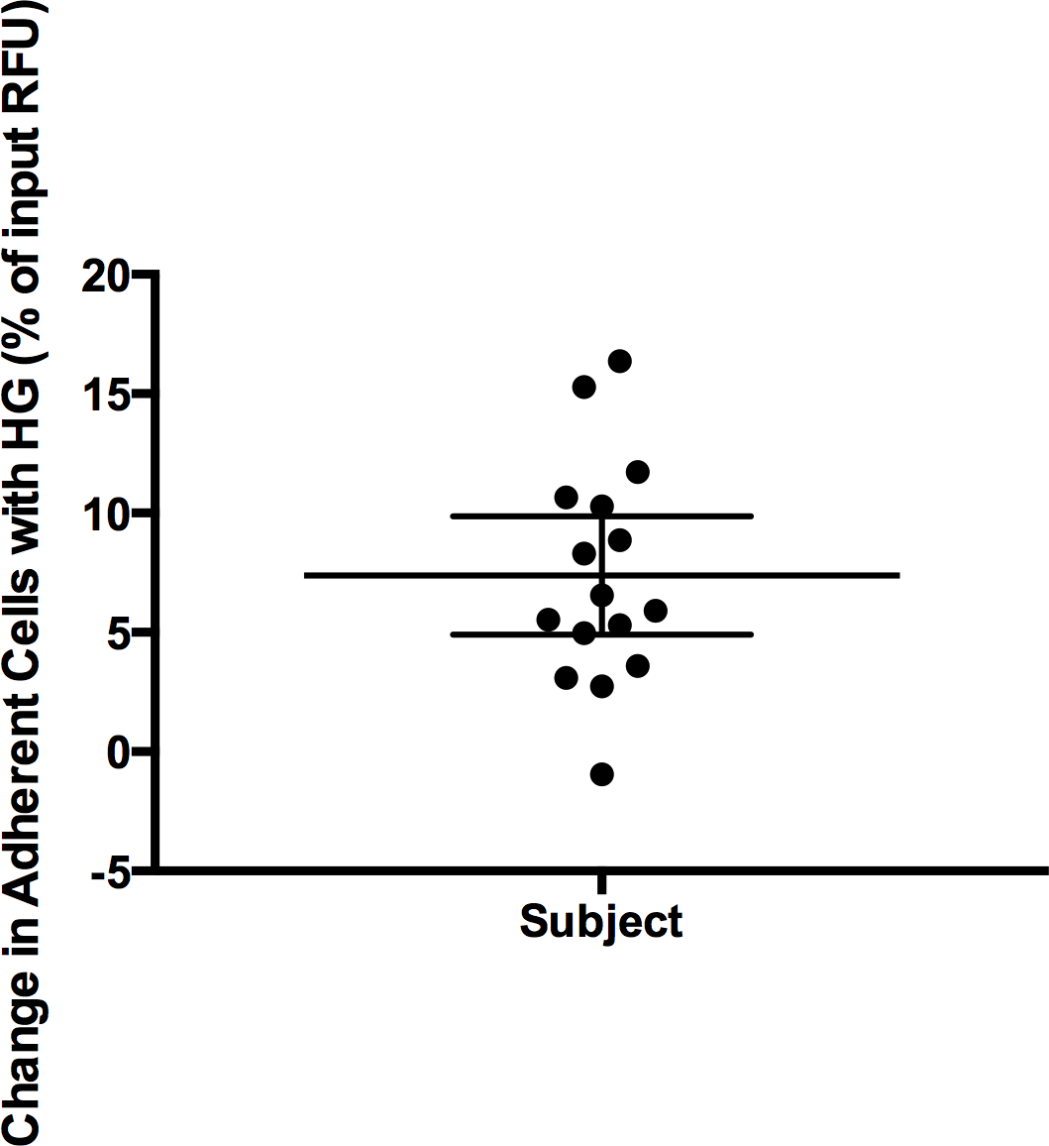
Leukocyte endothelial adhesion increases under high glucose conditions. Univariate scatter plot of the difference in adhesion for each of sixteen subject lymphoblastoid cell lines under high glucose (30 mM) (HG) compared to standard glucose (11 mM). A significant increase in adhesion is seen under high glucose conditions (p = 1.28x10^-5^). Change in adhesion reveals increased endothelial adhesion in high glucose. Mean and 95% confidence intervals shown.

Leukocyte endothelial adhesion was assessed in the sixteen DCCT/EDIC lymphoblastoid cell lines under standard and high glucose conditions. For this experiment a high throughput assay that we had previously developed was employed (Z′-factor = 0.67) [9]. Similar to our prior work we found the assay to be robust and reliable. Little variability was evident among runs as revealed by the low coefficients of variation (Table 1). A significant increase in adhesion was seen under high glucose conditions (p = 1.28x10^-5^) (Fig 3). We confirmed inter-individual differences among the cell lines for leukocyte endothelial adhesion with analysis of variance (F = 73.07, p-value < 0.0001). Of the total variance in leukocyte endothelial adhesion, 80% was cell line dependent. Hence, high glucose stimulated a cell line specific increase in leukocyte endothelial adhesion in lymphoblastoid cells.

**Table 1:** Leukocyte adhesion to human retinal microvascular endothelium is reliably assessed by high throughput assay.

		SG			HG	
cell line	Mean	stdev	cv%	Mean	stdev	cv%
1	44	4	9	47	3	7
2	17	1	8	22	2	7
3	22	1	5	33	2	7
4	36	3	8	47	4	8
5	34	3	8	39	4	10
6	46	3	6	52	2	5
7	36	1	4	36	3	7
8	24	1	5	29	9	31
9	18	1	6	29	2	6
10	27	3	10	42	2	6
11	18	2	13	26	1	4
12	18	2	12	21	0	2
13	17	1	5	26	2	9
14	23	2	8	39	2	5
15	20	1	6	27	1	5
16	11	0	4	15	1	5

Measurements performed in sixteen subject lymphoblastoid cell lines. Adhesion (n = 4) was measured under both standard glucose cell culture conditions (SG) (11 mM glucose) and high glucose (HG) conditions (30 mM glucose) for each lymphoblastoid cell line. Mean, standard deviation (stdev), and coefficient of variation in percent (cv%) are reported for each subject lymphoblastoid cell line and reflect the number of adherent cells as a % of the input in relative fluorescence units (RFU).

### High glucose increases the generation of reactive oxygen species

**Fig 4:**
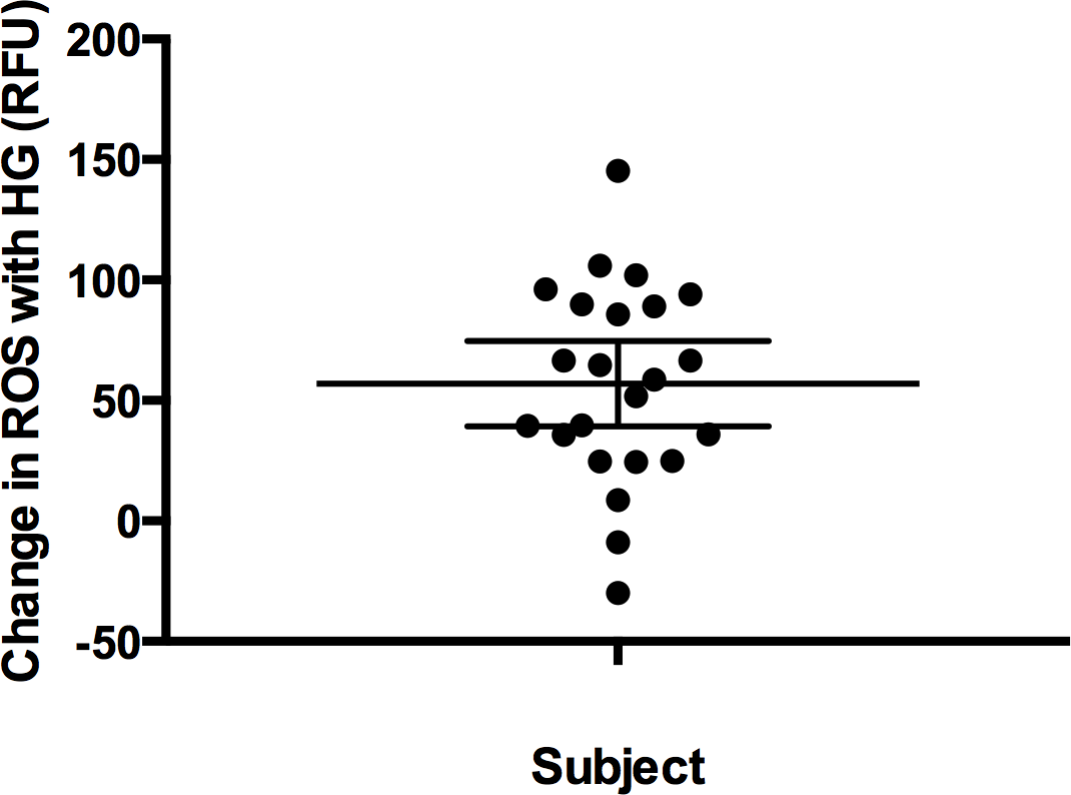
Reactive oxygen species are increased in lymphoblastoid cell lines exposed to high glucose. Measurements performed in twenty-three subject lymphoblastoid cell lines. Reactive oxygen species were measured under both standard (11 mM) and high glucose (HG) (30 mM) cell culture conditions for each lymphoblastoid cell line. Reactive oxygen species assayed by mean CM-H2DCFDA fluorescence and reported in relative fluorescence units (RFU). Univariate scatter plot demonstrates differential response for each of 23 subject lymphoblastoid cell lines to high glucose for the formation of reactive oxygen species. Significant increases in reactive oxygen species production in high glucose are evident (p = 2.56x10^–6^). Scatter plot reveals increased reactive oxygen species formation for most but not all lymphoblastoid cell lines in high glucose compared to standard glucose conditions. Mean and 95% confidence intervals shown.

Finally, we measured the generation of reactive oxygen species following high glucose stimulation in the twenty-three lymphoblastoid cell lines. Reactive oxygen species were assayed by mean CM-H2DCFDA fluorescence. An increase in reactive oxygen species was evident following high glucose (p = 2.56x10^−6^) (S6 Table). The differences among lines seemed to fall broadly in a bell curve distribution (Fig 4).

No differences in response were discernable between three clinical groups (subjects with diabetes and proliferative diabetic retinopathy, subjects with diabetes without diabetic retinopathy and non-diabetic control subjects) (S3 Fig). We assessed whether or not there was any significant difference between the three different groups based on gene expression [40], CD18 expression, leukocyte endothelial adhesion, and reactive oxygen species generation. After controlling for multiple measures no significant differences were identified (S7A Table). There was also no difference present when comparing the diabetic subjects alone (PDR vs No PDR) (S7B Table).

## Discussion

Progress in the treatment of diabetic retinopathy has been hindered by limited access to a biologically relevant tissue that is correlated to human clinical data. Human eye tissue is not routinely available for research purposes except from post-mortem specimens. Individual donors may not have diabetic retinopathy, and for those who do, detailed associated clinical records of their care may not be available. Problematic access to tissue for investigators has resulted in a proliferation of diabetic retinopathy models to simulate the human condition in animals such as mice, monkeys, fish, and cats. We investigated whether lymphoblastoid cell lines could be utilized for this purpose as they are available for many of the subjects of several large clinical epidemiologic studies of diabetic retinopathy, including the landmark Diabetes Control and Complications Trial/Epidemiology of Diabetes Interventions and Complications (DCCT/EDIC) cohort.

Initially, we investigated whether high glucose was capable of inducing a relevant biologic response in mature lymphoblastoid cells similar that seen in primary leukocytes. There were several reasons why we were uncertain whether this would be the case. First, lymphoblastoid cells are transformed. A major challenge in their use is that in a quiescent state mature lymphoblastoid cell lines assume a homogeneous gene expression profile that reflects a uniform transformed B cell state [39]. Second, they undergo years of storage and multiple freeze-thaw cycles. Third, they are derived from B-cells, which are not likely the primary pathogenic leukocyte in diabetic retinopathy. Earlier work has mostly implicated monocytes [41–43] and neutrophils [44, 45] as the key leukocyte classes involved in diabetic retinopathy [46], but studies of other cell types including B cells [47] and their relation to retinopathy are ongoing [48]. Finally, the standard glucose concentration in which lymphoblastoid cells are cultured is 11 mM, notably higher than physiologic levels. When cultured in physiologic 5 mM glucose it resulted in both poor growth and reduced viability of the lymphoblastoid cells.

On the other hand, we considered that as lymphoblastoid cell lines originate from white blood cells, at a minimum they should recapitulate many of their in vivo characteristics. Originally developed as a perpetual source of DNA, they have been shown to have a great deal of biologic relevance. Specifically, studies investigating gene regulation [49–56], gene knockdown [57], radiation response [58] and pharmacogenomics [59–62] have all recently been successfully conducted in lymphoblastoid cell lines [63, 64]. Furthermore, the translatability of lymphoblastoid cells has been demonstrated in multiple diseases despite their transformed nature. In particular, lymphoblastoid cells have served as an excellent model for those complex conditions in which white blood cells are the most relevant human tissue such as rheumatoid arthritis, type 1 diabetes, Crohn’s disease, and multiple sclerosis [8, 11, 65].

Circulating peripheral leukocytes have clearly been implicated in the pathogenesis of diabetic retinopathy [66–69]. Diabetes induces an intrinsic systemic inflammatory response [70, 71]. It is not unexpected then that an early feature of diabetic retinopathy is a presence of systemic inflammatory markers [72, 73]. Leukocytes are a key mediator of inflammation throughout the body. Leukocytes are activated in the diabetic state [41]. The key features of leukocytes that have been associated with the pathogenesis of diabetic retinopathy include differences in adhesion, gene/protein expression, and endothelial effects. Leukocytes have been shown to directly kill endothelial cells, and this is significantly worse when using leukocytes collected from diabetic mice or patients [46, 74, 75]. It is thought that endothelial injury in diabetes is mediated in part by enhanced leukocyte release of reactive oxygen species like superoxide, inflammatory metabolites, and contact mediated mechanisms [45, 46, 68, 73, 76–78]. Amelioration of leukocyte-endothelial adhesion reduces microvascular injury [79, 80]. Inhibition of leukocyte killing of vascular cells in preclinical animal models of diabetic retinopathy protects against diabetes-induced microvascular changes in the retina, demonstrating a key and primary role of peripheral, circulating leukocytes in diabetic retinopathy [46, 81–83]. Hence, we anticipated that lymphoblastoid cell lines would demonstrate similar molecular, cellular, and functional alterations to high glucose. By focusing on the differential cellular response to a net increase of 19 mM glucose (342 mg/dl), we were optimistic that the homeostatic adjustments induced in lymphoblastoid cells would mirror those that occur in vivo. Our prior work and that of other groups also suggests that cellular changes at these absolute concentrations are likely to generate a specific glucose-mediated effect, not affected by the increased osmolarity of the media [36, 84]. Indeed, we found that following chronic exposure to high glucose, lymphoblastoid cell lines revealed increased expression of genes that included cytokines, transcription factors, and adhesion molecules previously implicated in the pathogenesis of diabetic retinopathy (Fig 1) [66, 67, 73]. These results may have been even more dramatic in 5 mM baseline solution as opposed to the standard cell culture conditions of 11 mM glucose used in this study.

In a dynamic state, the vast majority of the cellular proteome is generated in response to transcriptional induction upon stimulation [85]. Hence, we hypothesized that stimulation of the lymphoblastoid cells with high glucose would result not only in the gene expression changes that we observed but also that these changes would be maintained at the protein level. The numerous successful eQTL mapping studies performed to date [53, 54, 86] demonstrate that these transcriptional differences, when present in lymphoblastoid cell lines, are valid and reflective of true underlying biology. In our study the effect of high glucose on transcription was maintained when protein expression was assessed. CD18, an adhesion molecule that showed a significant increase in gene expression to high glucose, similarly demonstrated elevated protein expression under high glucose conditions.

The increased expression of CD18 and other adhesion molecules resulted in a functional effect on the lymphoblastoid cells. Specifically, we observed an increased adhesiveness of lymphoblastoid cells to endothelial cells in our cell-based assay. Moreover, as we identified the up-regulation of several cytokines that have been shown to stimulate mitochondrial oxidative stress [87], we similarly reasoned that there would be a corresponding effect on the generation of reactive oxygen species in the lymphoblastoid cells. In fact, we found that generation of reactive oxygen species in lymphoblastoid cells was increased in the setting of high glucose. Hence, stimulation of lymphoblastoid cells with high glucose demonstrated corresponding changes on molecular, cellular and functional levels. Taken together these collective findings suggest that high glucose stimulation of lymphoblastoid cells results in profound changes reflective of those seen in primary diabetic leukocytes.

Next, we were interested in assessing whether lymphoblastoid cell lines reveal individual differential responses at the molecular and cellular level to high glucose. At baseline in a quiescent state mature lymphoblastoid cell lines assume a homogeneous transformed B cell phenotype [39]. In fact, the gene expression among mature lymphoblastoid cell lines is more alike than to the individual primary cell lines from which they were derived. If the transformed state is the primary determinant of cellular phenotype, then one might anticipate little difference in a high glucose state both in a given subject as well as among different subjects. In response to high glucose we observed an induction of transcription that resulted in corresponding and predictable downstream cellular and functional effects in the lymphoblastoid cells. As each cell line harbors the unique DNA signature of the individual subject and it is initially through gene expression that an individual’s genotype exerts its affects on phenotype, we hypothesized that the response to glucose would be cell line specific. For these reasons, we predicted that the glucose response would be heterogeneous. If this were the case, then there should be individual differences seen in the reaction to the same exogenous stimulus of high glucose among the twenty-three different lymphoblastoid cell lines. In this study, we found that high glucose was capable of distinguishing among individuals. In particular, when we evaluated the specific responses for gene expression, CD18 protein expression, endothelial adhesion, and reactive oxygen species generation for each of the lymphoblastoid cell lines, significant inter-individual differences were present. Thus, individual differential responses to the same provocative agent were evident in lymphoblastoid cells.

What these changes were unable to distinguish between, though, were the three groups of clinical subjects (composed of individuals without diabetes, individuals with diabetes and no retinopathy, and individuals with diabetes and proliferative diabetic retinopathy) (S7 Table). In hindsight, this is not surprising given the small sample size. While larger cohorts may help to realize differences between different clinical sub-groups, since the genetic contribution to these conditions is modest [88, 89], the range of variation produced in response to high glucose exposure will necessarily be modest. Common molecular responses on average to high glucose regardless of retinopathy status among the subject cell lines emphasize the key importance of degree and duration of glycemia as the primary risk factors for diabetic complications like retinopathy [90–92]. Since these non-genetic (environmental) factors play such a major, predominant role in dictating the development of diabetic complications, it has created a significant challenge in identifying their genetic basis [6, 93].

Exposure to uniform cell culture conditions that produce differences among individuals, though, can generate insights into the aberrant pathways that may underlie a particular patient’s disease. Differences in response among subjects should reflect differences at the level of the individual’s genome since all environmental factors are uniform [94, 95]. For diabetic complications, like retinopathy, these findings suggest that management of affected individuals may be best approached at the individual level. For example, the reactive oxygen species data (Fig 4) suggest that an anti-oxidant strategy might not be equally effective in all subjects with retinopathy. In fact, the widely divergent responses to high glucose in all study parameters for individuals with retinopathy emphasize the multifactorial nature of this condition and the potential importance of a personalized approach that takes into account these inter-individual differences, as is beginning to occur more frequently in cancer care. Lymphoblastoid cell lines may be an ideal tool for understanding this individual variability. They may offer a means to dissect the genetic and environmental interactions present in diabetic complications thereby accelerating the transition to more targeted, patient-specific treatments.

## Supporting Information

S1 Fig Proportion of variance in gene expression by covariate. Figure is variance component analysis of gene expression. It demonstrates that differences in the individual subject lymphoblastoid cell line gene expression response to high glucose explain most of the inter-subject variance.
(TIFF)

S2 Fig Fold Change In Gene Expression From SG to HG. Principal component analysis of the twenty-three samples did not reveal clustering of any of the three groups when differences in gene expression were compared between the three groups. Subjects without diabetes (Red—No DM). Subjects with diabetes but no retinopathy (Green–No PDR). Subjects with diabetes and proliferative diabetic retinopathy (Blue–PDR).
(PDF)

S3 Fig **(A-I) No differences were identified in response to high glucose between the three clinical groups.** Figure shows univariate scatter plots of response to high glucose (HG) for subjects without diabetes (No DM), with diabetes and no diabetic retinopathy (No DR), and with proliferative diabetic retinopathy (PDR). Differences in gene expression (Figures A-F in S3), protein expression (CD18) (Figure G in S3), leukocyte endothelial adhesion (No DR vs PDR only) (Figure H in S3) and reactive oxygen species (Figure I in S3) are shown for each cell line comparing its response in standard and high glucose conditions.
(PDF)

S1 Table DCCT/EDIC Subjects. (PDF)

S2 Table Coriell Institute for Medical Research NIGMS Human Genetic Cell Repository subject lymphoblastoid cell lines. (PDF)

S3 Table Pre-validated qPCR primers. (PDF)

S4 Table Gene expression data for each of the twenty-three subjects in both standard glucose (SG) and high glucose (HG). ct cycle threshold.
(PDF)

S5 Table CD18 expression by flow cytometry. Protein expression for each of the twenty-three subjects in both standard glucose (SG) and high glucose (HG). Units are in relative fluorescence (RFU).
(PDF)

S6 Table Reactive oxygen species generation. Reactive oxygen species were measured under both standard glucose cell culture conditions (SG) (11 mM glucose) and high glucose (HG) conditions (30 mM glucose) for each lymphoblastoid cell line. Reactive oxygen species were assayed by mean CM-H2DCFDA fluorescence.
(PDF)

S7 Table Comparison between clinical groups in response to high glucose. a) Proliferative diabetic retinopathy (PDR), diabetes without retinopathy (No DR), and no diabetes (No DM). b) DCCT/EDIC Participants: No DR vs PDR.
(PDF)
