# Supplementary figures and images for: Lymphoblastoid Cell Lines as a Tool to Study Inter-Individual Differences in the Response to Glucose
Source: PLoS One. 2016 Aug 10;11(8):e0160504. doi: 10.1371/journal.pone.0160504 (PMC4979894; doi:10.1371/journal.pone.0160504)

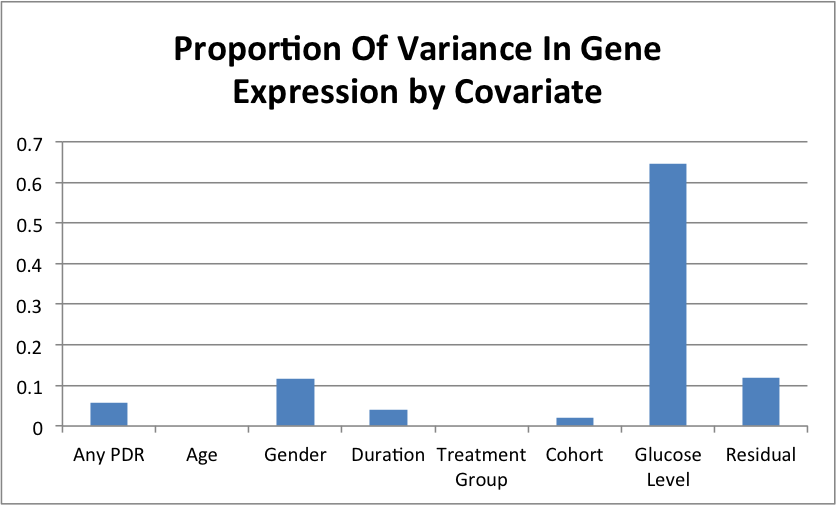

Supplement: S1 Fig — Figure is variance component analysis of gene expression. It demonstrates that differences in the individual subject lymphoblastoid cell line gene expression response to high glucose explain most of the inter-subject variance. (TIFF) [file pone.0160504.s001.tiff]

PC2 (20.5% explained var.)

5.0  
2.5  
0.0  
-2.5  
-5.0

-4

-2

0

2

PC1 (56.0% explained var.)

**groups**

No DM

No PDR

PDR

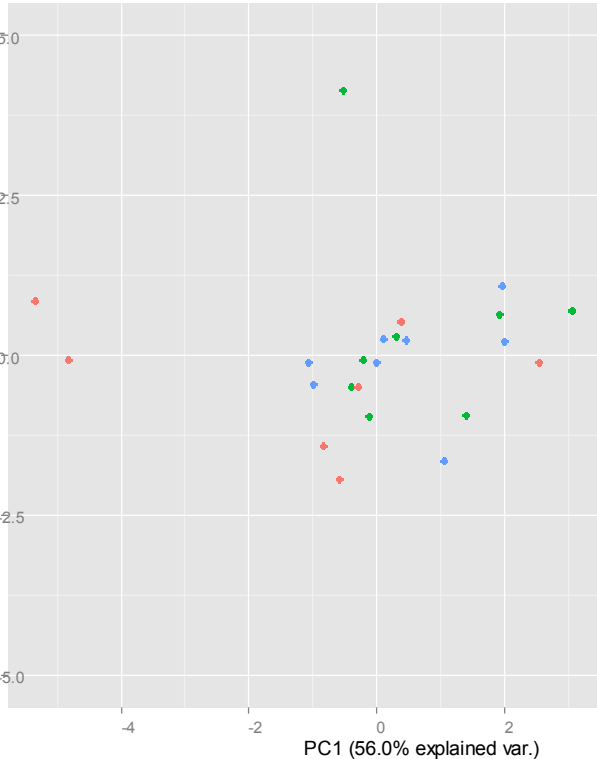

Supplement: S2 Fig — Principal component analysis of the twenty-three samples did not reveal clustering of any of the three groups when differences in gene expression were compared between the three groups. Subjects without diabetes (Red—No DM). Subjects with diabetes but no retinopathy (Green–No PDR). Subjects with diabetes and proliferative diabetic retinopathy (Blue–PDR). (PDF) [file pone.0160504.s002.pdf]

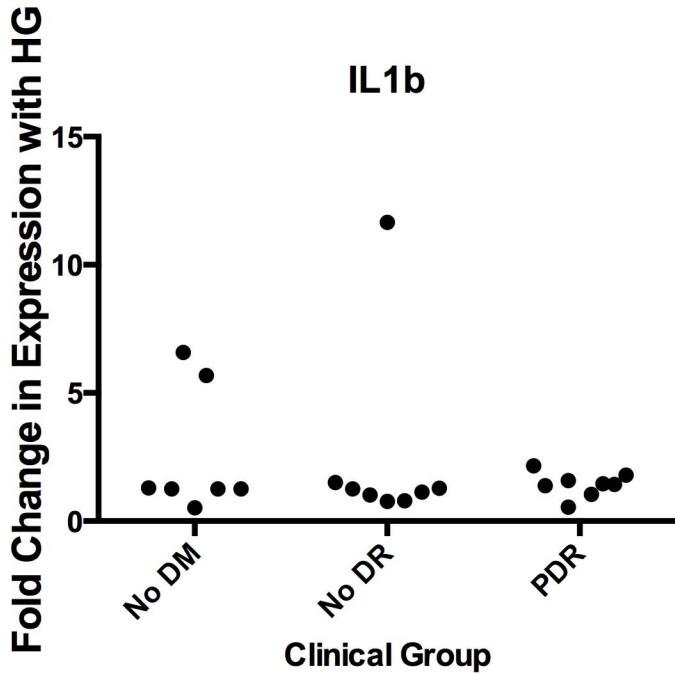

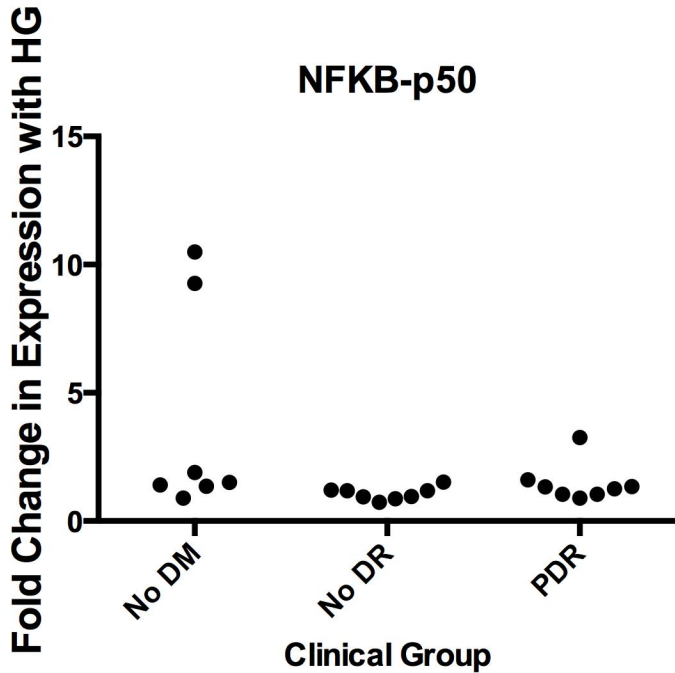

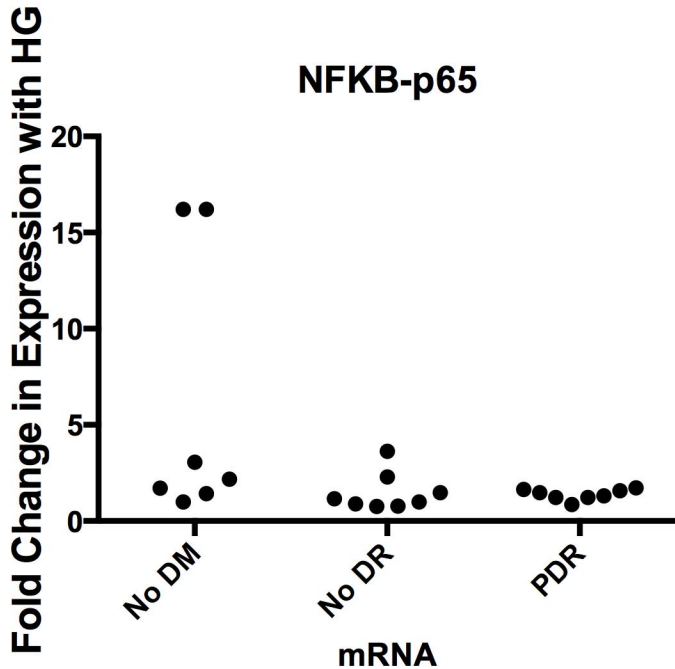

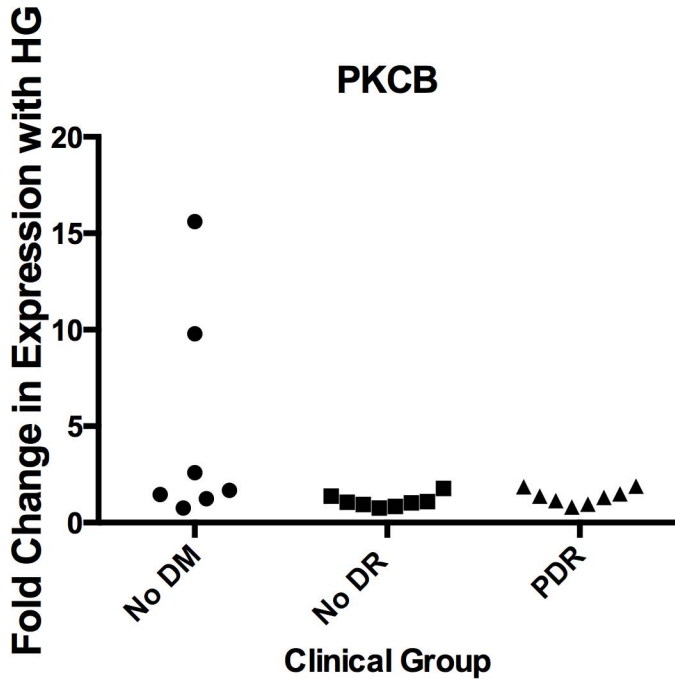

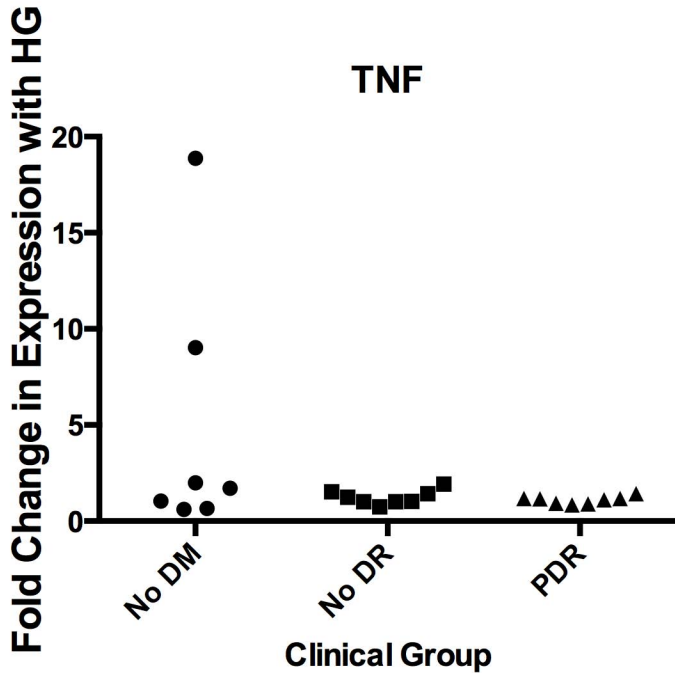

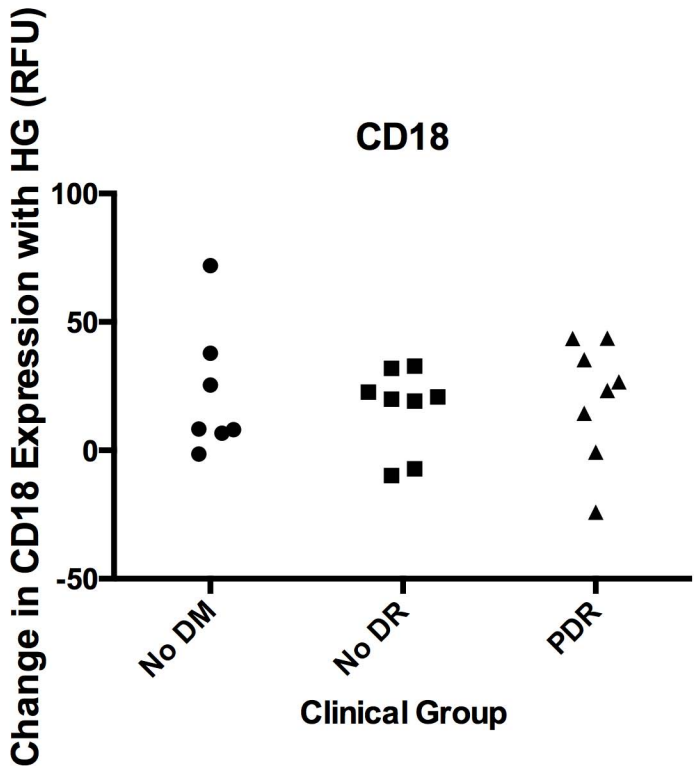

## Leukocyte Endothelial Adhesion

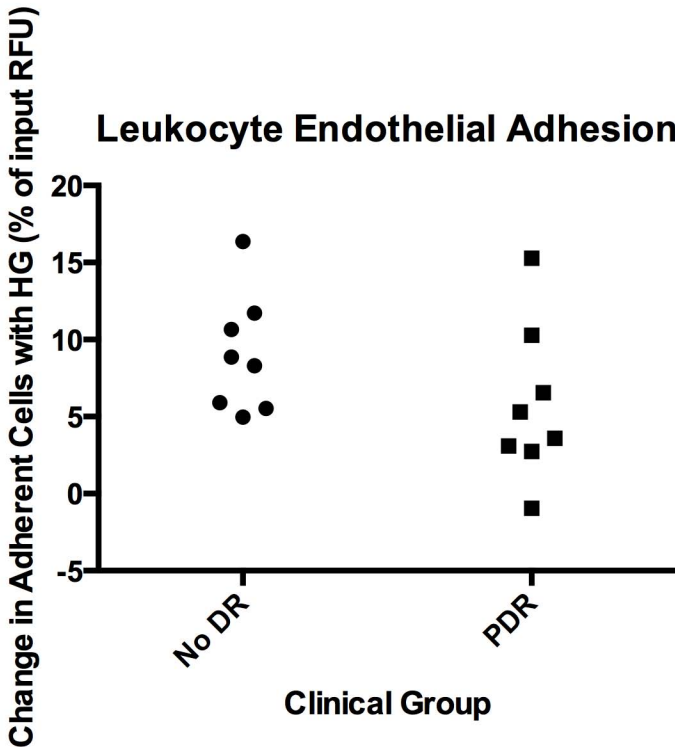

# Reactive Oxygen Species

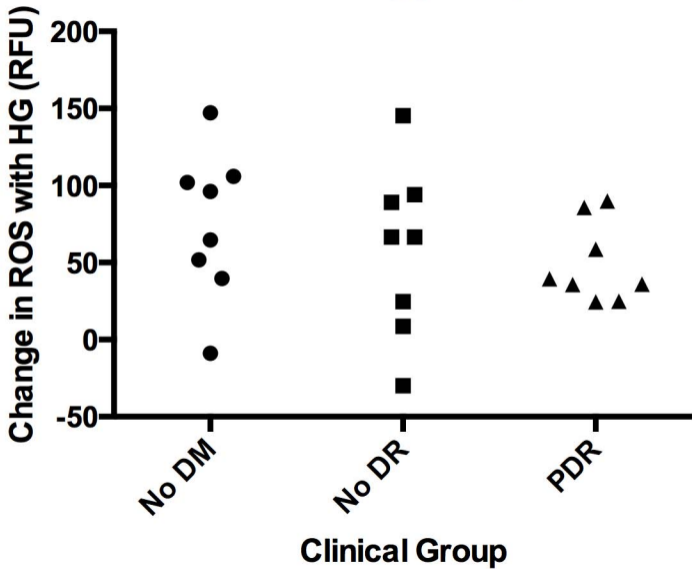

Supplement: S3 Fig — (A-I) No differences were identified in response to high glucose between the three clinical groups. Figure shows univariate scatter plots of response to high glucose (HG) for subjects without diabetes (No DM), with diabetes and no diabetic retinopathy (No DR), and with proliferative diabetic retinopathy (PDR). Differences in gene expression (Figures A-F in S3), protein expression (CD18) (Figure G in S3), leukocyte endothelial adhesion (No DR vs PDR only) (Figure H in S3) and reactive oxygen species (Figure I in S3) are shown for each cell line comparing its response in standard and high glucose conditions. (PDF) [file pone.0160504.s003.pdf]
