# Supplementary material for: Lymphoblastoid Cell Lines as a Tool to Study Inter-Individual Differences in the Response to Glucose
Source: PLoS One. 2016 Aug 10;11(8):e0160504. doi: 10.1371/journal.pone.0160504 (PMC4979894; doi:10.1371/journal.pone.0160504)
Supplement: S1 Table — (PDF) [file pone.0160504.s004.pdf]

S1 Table: DCCT/EDIC Subjects

	Diabetes with No Diabetic Retinopathy	Proliferative Diabetic Retinopathy
N	8	8
Mean age in years at baseline* (std)	30 (6.0)	31 (8.5)
Caucasian ethnicity (%)	100	100
Mean duration of type 1 diabetes in months at baseline (std)	26 (12.7)	53 (43.4)
Female (%)	5 (62.5)	5 (62.5)
Intensive Treatment Group (%)^	3 (37.5)	3 (37.5)
Secondary Intervention Cohort (%)#	0 (0)	1 (12.5)

* DCCT baseline at subject enrollment (1983–1989); (std) standard deviation

^ For the duration of the DCCT study the intensive treatment group maintained a HbA1c of about 7% as compared to 9% in the conventional treatment group.

#The Secondary Intervention Cohort consisted of subjects with type 1 diabetes for 1-15 years and mild to moderate non-proliferative retinopathy and a urinary albumin excretion rate < 200 mg/dy. The Primary Prevention cohort consisted of subjects with type 1 diabetes for 1-5 years and no diabetes related complications.
