# Supplementary material for: Lymphoblastoid Cell Lines as a Tool to Study Inter-Individual Differences in the Response to Glucose
Source: PLoS One. 2016 Aug 10;11(8):e0160504. doi: 10.1371/journal.pone.0160504 (PMC4979894; doi:10.1371/journal.pone.0160504)
Supplement: S2 Table — (PDF) [file pone.0160504.s005.pdf]

S2 Table: Coriell Institute for Medical Research NIGMS Human Genetic Cell Repository subjects

Subject	Ethnicity	Gender	Age* (years)	BMI	Notes
GM14581	Caucasian	Male	18	24	
GM14569	Caucasian	Male	24		
GM14381	Caucasian	Female	20	21	#
GM07012	Caucasian	Female	N/A	N/A	CEPH%
GM14520	Caucasian	Female	22	32	
GM11985	Caucasian	Female	N/A	N/A	CEPH%
GM07344	Caucasian	Female	N/A	N/A	CEPH%

*At time of sampling
family history of diabetes
% Repository Linkage Families
N/A Not Available
