# Supplementary material for: Lymphoblastoid Cell Lines as a Tool to Study Inter-Individual Differences in the Response to Glucose
Source: PLoS One. 2016 Aug 10;11(8):e0160504. doi: 10.1371/journal.pone.0160504 (PMC4979894; doi:10.1371/journal.pone.0160504)
Supplement: S3 Table — (PDF) [file pone.0160504.s006.pdf]

S3 Table. **Pre-validated qPCR primers.**

No	Gene	Prime Time Primer Sequence
1	*GAPDH*<br>Hs.PT.39a.22214836	ACA TCG CTC AGA CAC CAT G<br>TGT AGT TGA GGT CAA TGA AGG G
2	*TNF*<br>Hs.PT.58.45380900	TGC ACT TTG GAG TGA TCG G<br>TCA GCT TGA GGG TTT GCT AC
3	*IL1B*<br>Hs.PT.58.39765191	GGA CAA GCT GAG GAA GAT GC<br>GAT CGT ACA GGT GCA TCG TG
4	*ITGB2/CD18*<br>Hs.PT.58.40013588	TGA AAC CCA GGA AGA CCA C<br>AGG TCC ATC AGA TAG TAC AGG T
5	*NFKB1*<br>Hs.PT.58.20380809	AGA AGT CTT ACC CTC AGG TCA<br>CAG TTA CAG TGC AGA TCC CA
6	*RELA/P65*<br>Hs.PT.58.27913791	GAA TGG CTC GTC TGT AGT GC<br>GCT GCT CAA TGA TCT CCA CAT
7	*PRKCB*<br>Hs.PT.58.20837947	GAA TGT TCC CAG CCT GTG T<br>GGA TCT GAC AGG CCA TTG G

IDT Coralville, Iowa, USA.
