# Supplementary material for: Lymphoblastoid Cell Lines as a Tool to Study Inter-Individual Differences in the Response to Glucose
Source: PLoS One. 2016 Aug 10;11(8):e0160504. doi: 10.1371/journal.pone.0160504 (PMC4979894; doi:10.1371/journal.pone.0160504)
Supplement: S4 Table — ct cycle threshold. (PDF) [file pone.0160504.s007.pdf]

S4 Table: Gene Expression

	TNF		*IL1B*		*NFKB-p50*		*NFKB-p65*		*CD18*		*PKCB*		*GAPDH*	
	SG	HG	SG	HG	SG	HG	SG	HG	SG	HG	SG	HG	SG	HG
**cell line**	Δct	Δct	Δct	Δct	Δct	Δct	Δct	Δct	Δct	Δct	Δct	Δct	Δct	Δct
1	5.30	5.48	13.83	12.72	5.12	5.07	8.95	9.18	5.09	5.25	5.62	5.94	0	-0.82
2	6.62	5.67	11.06	11.04	6.32	6.08	10.31	10.48	7.95	7.20	6.93	6.81	0	1.02
3	6.03	6.01	13.24	12.89	5.49	5.70	9.78	9.79	6.76	6.83	5.60	5.86	0	0.73
4	6.67	6.92	13.31	14.21	6.69	6.62	11.35	10.78	7.56	7.04	6.97	6.59	0	0.18
5	6.49	6.45	12.70	12.11	6.30	5.70	11.11	9.91	6.75	6.42	6.89	6.44	0	0.39
6	5.61	5.09	12.34	8.80	6.03	6.08	10.20	10.61	6.75	6.77	6.49	5.67	0	5.70
7	5.68	5.45	14.31	13.78	6.03	5.70	10.34	9.63	5.93	5.75	6.57	6.12	0	0.16
8	6.49	6.61	14.11	14.06	4.85	5.00	9.00	8.60	3.92	4.38	4.25	4.32	0	0.41
9	7.49	6.88	14.36	14.04	6.87	6.63	11.31	11.10	8.78	7.06	6.40	6.37	0	1.56
10	5.48	5.34	12.48	11.82	5.99	5.57	10.21	9.91	6.27	6.18	6.46	5.88	0	0.95
11	5.35	5.33	10.58	10.92	5.57	6.03	9.93	10.29	6.46	6.68	5.73	6.12	0	1.59
12	7.21	6.97	14.93	14.46	6.83	6.42	10.88	10.58	7.33	7.31	7.21	7.03	0	0.81
13	5.09	5.52	11.57	11.95	5.66	5.72	10.18	9.62	6.09	5.74	6.13	6.04	0	-0.55
14	6.25	5.93	12.20	12.02	6.55	6.27	12.15	10.30	7.87	7.15	6.60	6.70	0	-1.43
15	3.54	3.33	11.31	10.79	4.61	2.91	8.26	7.48	4.67	4.52	4.25	3.33	0	-1.28
16	7.72	7.21	15.60	14.75	7.06	6.38	11.17	10.52	7.66	6.94	7.02	6.13	0	0.70
14381 GM	6.47	5.48	15.27	16.22	6.02	5.52	10.49	9.36	7.14	6.02	6.84	6.30	0	1.18
14569 GM	7.67	6.91	9.21	8.89	6.39	5.94	10.73	10.74	7.55	7.59	5.74	5.43	0	0.47
7344 GM	5.65	2.48	12.80	10.29	5.48	2.09	10.02	6.00	6.24	2.39	5.89	2.60	0	4.29
7012 GM	7.70	3.47	10.97	8.25	6.57	3.36	11.23	7.21	7.94	3.84	6.81	2.85	0	5.29
14520 GM	6.13	6.08	13.98	13.66	7.00	6.41	11.40	10.64	6.98	6.71	7.21	6.46	0	-3.63
14581 GM	5.56	6.31	13.34	12.96	6.28	6.44	10.29	9.79	6.26	6.47	6.45	6.83	0	-3.31
11985 GM	6.08	6.68	15.29	14.97	7.18	6.25	11.53	9.92	7.28	6.45	7.39	6.02	0	-3.50
