# Supplementary material for: Lymphoblastoid Cell Lines as a Tool to Study Inter-Individual Differences in the Response to Glucose
Source: PLoS One. 2016 Aug 10;11(8):e0160504. doi: 10.1371/journal.pone.0160504 (PMC4979894; doi:10.1371/journal.pone.0160504)
Supplement: S5 Table — Protein expression for each of the twenty-three subjects in both standard glucose (SG) and high glucose (HG). Units are in relative fluorescence (RFU). (PDF) [file pone.0160504.s008.pdf]

S5 Table: CD18 expression (RFU)

cell line	SG	HG
1	154.12	189.39
2	206.86	199.7
3	152.89	184.77
4	175	218.53
5	173.19	195.84
6	190.24	223.11
7	160.94	184.26
8	145.1	188.8
9	197.26	216.46
10	182.45	209.09
11	200.25	220.16
12	177.11	153.07
13	219.72	209.89
14	196.04	216.89
15	203.78	203.13
16	200.39	214.86
GM14581	188.83	187.33
GM14569	191.54	229.4
GM14381	169.24	194.72
GM07012	244.37	251.07
GM14520	192.78	201.1
GM11985	203.75	211.79
