# Supplementary material for: Lymphoblastoid Cell Lines as a Tool to Study Inter-Individual Differences in the Response to Glucose
Source: PLoS One. 2016 Aug 10;11(8):e0160504. doi: 10.1371/journal.pone.0160504 (PMC4979894; doi:10.1371/journal.pone.0160504)
Supplement: S6 Table — Reactive oxygen species were measured under both standard glucose cell culture conditions (SG) (11 mM glucose) and high glucose (HG) conditions (30 mM glucose) for each lymphoblastoid cell line. Reactive oxygen species were assayed by mean CM-H2DCFDA fluorescence. (PDF) [file pone.0160504.s009.pdf]

S6 Table: Reactive Oxygen Species

cell line	ROS (RFU) SG	HG
1	91.17	175.53
2	109.92	134.42
3	137.19	204.76
4	76.05	141.28
5	71.99	137.42
6	180.47	195.02
7	140.53	162.63
8	91.88	132.78
9	147.67	241.04
10	163.4	194.18
11	203.26	207.91
12	134.73	168.86
13	119.07	215.84
14	118.5	271.85
15	161.87	168.56
16	89.55	184.53
GM14581	89.21	128.23
GM14569	148.16	243.36
GM14381	115.74	232.45
GM07012	245.33	220.95
GM14520	133.67	152.11
GM11985	133.83	234.18
GM07344	169.98	221.8
