# Supplementary material for: Lymphoblastoid Cell Lines as a Tool to Study Inter-Individual Differences in the Response to Glucose
Source: PLoS One. 2016 Aug 10;11(8):e0160504. doi: 10.1371/journal.pone.0160504 (PMC4979894; doi:10.1371/journal.pone.0160504)
Supplement: S7 Table — a) Proliferative diabetic retinopathy (PDR), diabetes without retinopathy (No DR), and no diabetes (No DM). b) DCCT/EDIC Participants: No DR vs PDR. (PDF) [file pone.0160504.s010.pdf]

S7 Table: Comparison between subjects

a) Proliferative diabetic retinopathy (PDR), diabetes without retinopathy (No DR), and no diabetes (No DM).

**Measure**	**HG-NG difference P-Value***
TNF	0.7994
IL1B	0.5658
NFKb P-50	0.0473
NFKb P-65	0.1413
CD18	0.4600
PKCB	0.1816
CD18 prot	0.7287
ROS	0.3159
LEA^	0.1953

*P-values generated using the Kruskal-Wallis test, using Monte-Carlo resampling for an approximation of the exact P-value.

^LEA P-values generated using exact Wilcoxon signed rank test.

Threshold p-value </= 0.125.

b) DCCT/EDIC Participants: PDR vs No DR

Measure	HG-NG difference P-Value*
TNF	0.4609
IL1B	1
NFKb P-50	0.1953
NFKb P-65	0.7422
CD18	0.3125
PKCB	0.2049
CD18 prot	0.3828
ROS	0.6406
LEA	0.1953

*P-values generated using exact Wilcoxon signed rank test.
